# A Phenotype and Genotype Case Report of a Neonate With Congenital Bilateral Coronary Artery Fistulas and Multiple Collateral Arteries

**DOI:** 10.3389/fcvm.2022.939551

**Published:** 2022-07-06

**Authors:** Shixin Su, Shuliang Xia, Ye He, Jianbin Li, Li Ma, Xinxin Chen, Jia Li

**Affiliations:** ^1^Clinical Physiology Laboratory, Institute of Pediatrics, Guangzhou Women and Children's Medical Center, Guangzhou Medical University, Guangdong, China; ^2^Guangdong Provincial Key Laboratory of Research in Structural Birth Defect Disease, Guangzhou Women and Children's Medical Center, Guangzhou Medical University, Guangdong, China; ^3^Cardiovascular Surgery, Heart Center, Guangzhou Women and Children's Medical Center, Guangzhou Medical University, Guangdong, China; ^4^Department of Pediatric Surgery, Institute of Pediatrics, Guangzhou Women and Children's Medical Center, Guangzhou Medical University, Guangdong, China; ^5^Cardiac Intensive Care Unit, Heart Center, Guangzhou Women and Children's Medical Center, Guangzhou Medical University, Guangdong, China

**Keywords:** congenital heart defect, coronary artery fistula, multiple collateral arteries, *DRC1*, whole-exome sequencing

## Abstract

We report a unique case of an 18-day-old girl with three coronary artery fistulas to the right atrium and right ventricle, respectively: three collateral arteries arising from the descending aorta and one from the right subclavian artery draining through a sac to the top of the right atrium, patent ductus arteriosus, and atrial septal defect. She presented symptoms of acute congestive heart failure. Cardiac catheterization and surgical interventions were performed to repair the defects. The patient recovered uneventfully and grew up well at 3 years of follow-up. Whole-genome sequencing (WES) in the patient, compared to her parents, showed 17 variants within 11 genes. Among these, only compound heterozygous mutation, c.T470G (p.L157R) and c.A1622G (p.D541G), in the *DRC1* gene have been reportedly related to congenital heart disease and are the most likely causative in our patient.

## Introduction

Congenital coronary artery fistulas account for 0.4% of congenital heart defects (CHD) (1). Coronary artery fistulas arising from the right coronary artery are present in 50–60% of cases, from the left anterior descending coronary artery in 25–40%, and both coronary arteries in 5% (2–4). Coronary artery fistulas terminate most frequently in the right ventricle, followed by the right atrium, coronary sinus, and pulmonary arterial trunk (2, 5, 6). Children with such a defect are generally asymptomatic, but congestive heart failure may happen in those with a substantial left to right shunt (7). About 55–80% of coronary arterial fistulas are isolated and 20–45% are associated with other CHD, such as patent ductus arteriosus, atrial, and ventricular septal defects (3).

Major collateral arteries are usually a component of cyanotic congenital heart defects that are embryologically linked with pulmonary valve atresia or near atresia (8, 9), clinically called MAPCAs, i.e., major aortopulmonary collateral arteries. These collateral arteries regress normally with the formation and development of pulmonary arteries but may persist in pulmonary atresia or hypoplasia (10). Congenital collateral arteries are rare. To our knowledge, there has been only one report of an association of coronary arterial fistula with an arteriovenous collateral artery to the superior vena cava (11).

The etiology of CHD remains largely unknown, likely involving genetics, epigenetics, and environmental etiologies (12). The identification of gene mutation underlying CHD may improve our understanding of cardiovascular development. With WES, the list of known CHD genes is rapidly expanding. For example, Zaidi et al. (13) analyzed the 362 parent-offspring trios with the patient affected by CHD and found that *de novo* mutations in ~400 genes could account for about 10% of the CHD cases.

In this report, we described a unique phenotype and genotype case of congenital bilateral coronary artery fistulas with multiple collateral arteries from the aorta to the right atrium, patent ductus arteriosus, and atrial septal defect with genetic findings from the child and her parents.

## Case Report

The report was approved by the institutional Research Ethics Board at the Guangzhou Women and Children's Medical Center (No. 24801) and written informed consent was obtained.

### Clinical Details

An 18-day-old girl who presented with tachypnea, tachycardia, and poor feeding was admitted to the Cardiac Intensive Care Unit. She was delivered by caesarian section at 39-week gestation with a birth weight of 2.9 kg. She was the only child in the family. Her parents, as well as family and relatives, have been unaffected by CHD or other congenital abnormalities (Supplementary Table S1). At a physical examination, she had a heart rate of 145 beats /min, respiratory rate of 45 times /min, and blood pressure of 74/40 mmHg. An electrocardiograph showed sinus tachycardia and right ventricular hypertrophy. Using transthoracic echocardiography, she was diagnosed with bilateral coronary artery fistulas, multiple collateral arteries from the descending aorta, patent ductus arteriosus, atrial septal defect, and moderate mitral regurgitation and mild-moderate tricuspid regurgitation.

**Figure 1:**
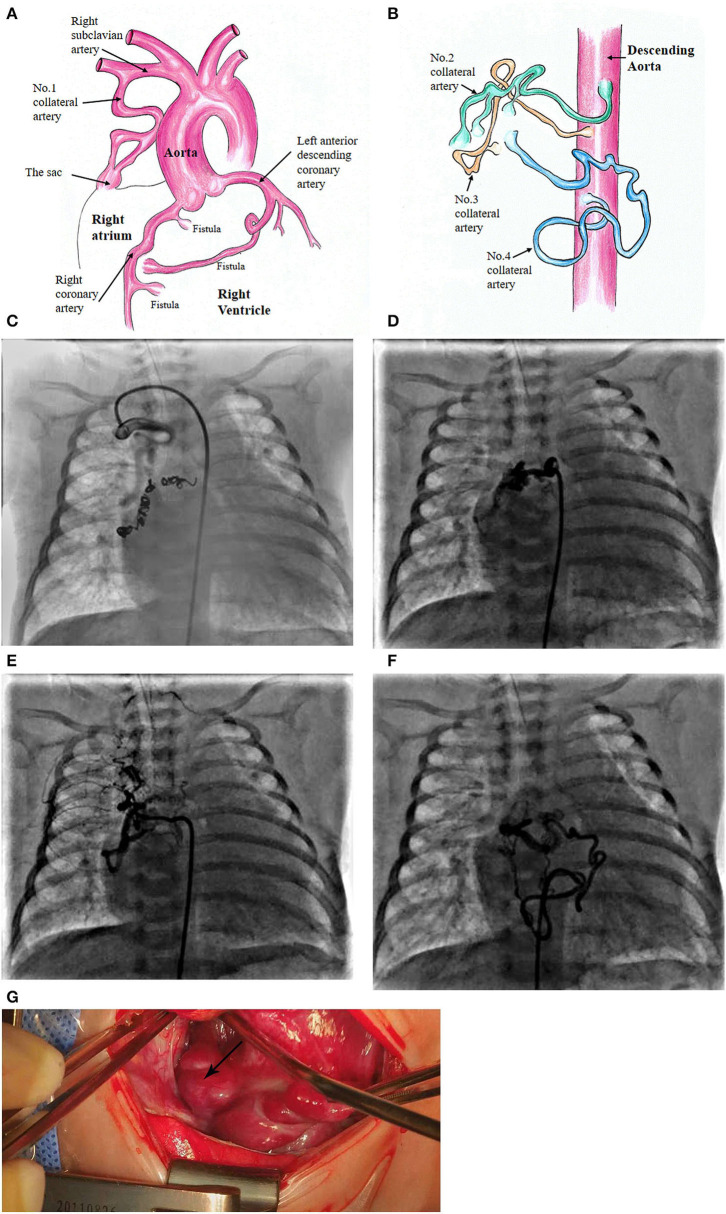
Schematic diagram of the patient's diagnosis of 3 coronary artery fistulas to the right atrium and right ventricle, respectively **(A)**. Four collateral arteries draining through a sac to the top of the right atrium **(A,B)**. Super-selective angiogram shows the following collaterals terminating to the sac and then to the right atrium. No. 1 collateral artery originating from the right subclavian artery **(C)**. No. 2 at the level of the 6th thoracic vertebra **(D)**. No. 3 originating from the descending aorta **(E)**. No. 4 located at the lower edge of the 9th thoracic vertebra **(F)**. Surgical image of the sac at the top of the right atrium **(G)**.

**Figure 2:**
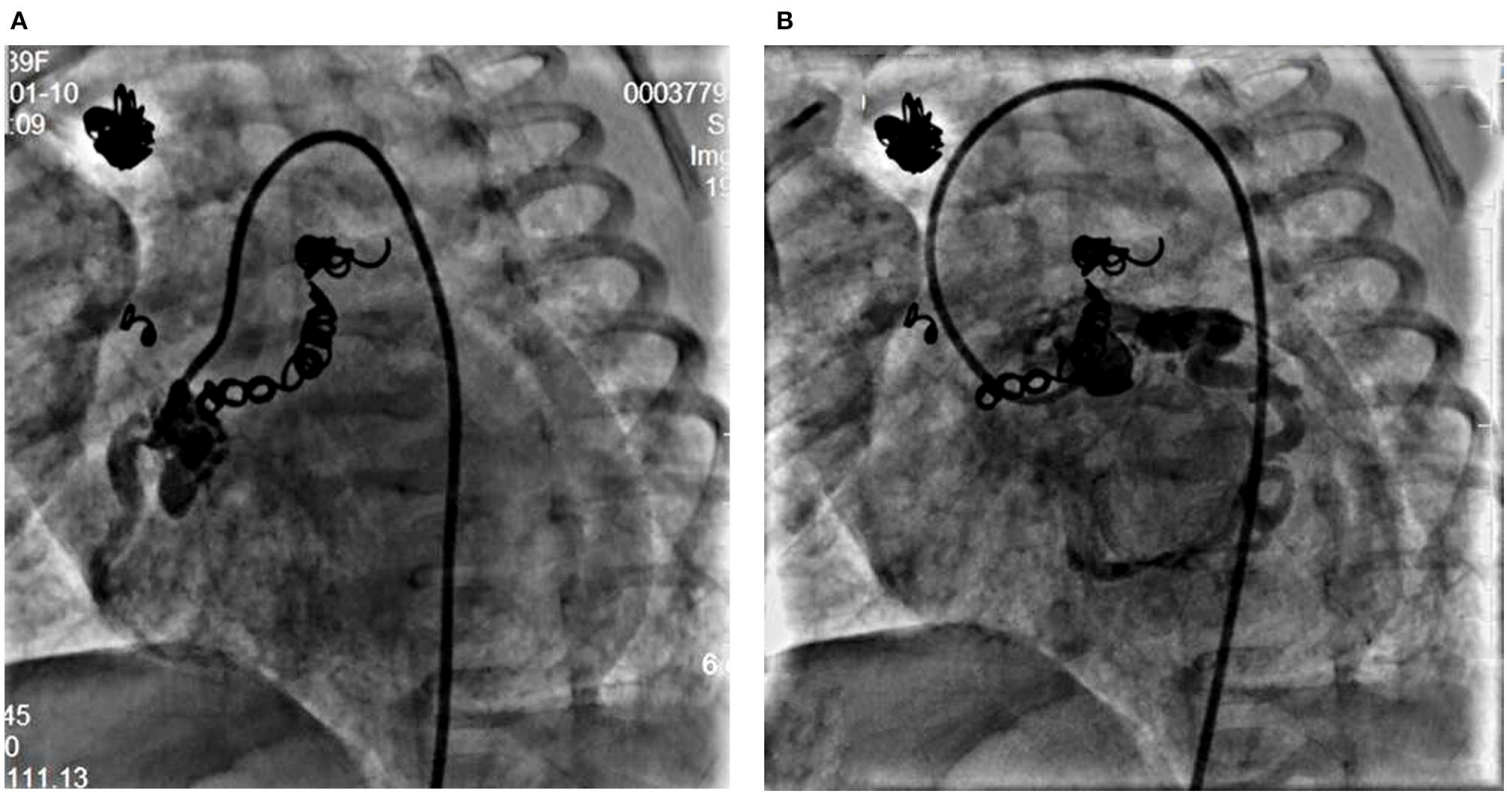
Right coronary angiogram from left anterior oblique 45° view shows the dilated right coronary artery fistula with a proximal fistula to the right atrium and a distal fistula into the right ventricle **(A)**. Left coronary angiogram shows a fistula from the left anterior descending coronary artery to the right atrium **(B)**.

At the age of 5 weeks old, cardiac catheterization was performed for hemodynamic assessment and interventions. The pulmonary to systemic blood flow ratio was 4.29 with pulmonary vascular resistance 1.7 Wood unit ^*^m^2^. A selective angiography revealed four collateral arteries draining into the right atrium. No. 1 (4.3 mm in diameter) of the collateral arteries was originating from the right subclavian artery (Figures 1A,C). No. 2 was seen at the level of the 6th thoracic vertebra and twisted to the right atrium (Figures 1B,D). No. 3 was a tortuous collateral artery (2.7 mm at the proximal and 1.7 mm at the minimal), located between the 6th and the 7th thoracic vertebra (Figures 1B,E). No. 4 was a very tortuous collateral artery between the descending aorta and the right atrium (Figures 1B,F). No. 1 and No. 3 collaterals were occluded with coils. Coronary arterial angiography also revealed a right coronary arterial fistula to the right ventricle and another to the right atrium (Figures 1A, 2A), and a fistula branching from the left anterior descending coronary artery to the right atrium (Figures 1A, 2B). Pulmonary arterial pressure was 43/22 mmHg.

More details were found at cardiac surgery at the age of 6 weeks old. A 15-mm sac was found an opening to the top of the right atrium (Figures 1A,G). Three collateral arteries originated from the anterior and posterior walls of the descending aorta and another from the right subclavian artery coursed into the sac and then the right atrium. The patent ductus arteriosus (4 mm) was ligated and cut down. Subsequently, a cardiopulmonary bypass was performed to suture the openings of the sac and the coronary arterial fistulas. The remaining two collateral arteries were clipped.

She had a smooth postoperative recovery and was discharged from the hospital on the 22nd postoperative day. At the 3rd year follow-up, the girl did not have any symptoms of congestive failure and grew up well.

### Genetic Analysis

Whole-exome sequencing (WES) was performed on the patient and her parents to screen for potentially causative mutations. Genomic DNA (gDNA) was extracted from peripheral blood samples. Exons were captured by the Agilent SureSelect Human All Exon V6 kit (Agilent, Santa Clara, CA), followed by high-throughput sequencing on the Illumina Hiseq 2500 platform to generate 150-bp paired-end reads. The average sequencing depth for the targeted region achieved 121x, and ~98% of targeted regions were covered at >20x. The sequencing of raw data was analyzed with Genome Analysis Toolkit (GATK) for removing low-quality, including adapter contaminated reads, N-contaminated reads with more than 10% bases, and reads with more than 55% low-quality bases (<5), and then, were aligned to the reference human genome (GRCh37/hg19) with Burrows-Wheeler Aligner (BWA). Sambamba was used to mark and remove duplicate reads. Single nucleotide variants (SNVs) and deletion/ insertions (indels) were called with SAMtools. The raw calls of SNVs and indels were filtered further with the following inclusion thresholds: (1) read depth > 5; (2) Root-Mean-Square mapping quality of covering reads > 30; and (3) the variant quality score of > 20. Finally, the variants were annotated with ANNOVAR. Annotations included minor allele frequencies (MAF ≤ 1%) from public control data sets, as well as deleteriousness and conservation scores, enabling further filtering and assessment of the likely pathogenicity of variants.

**Figure 3:**
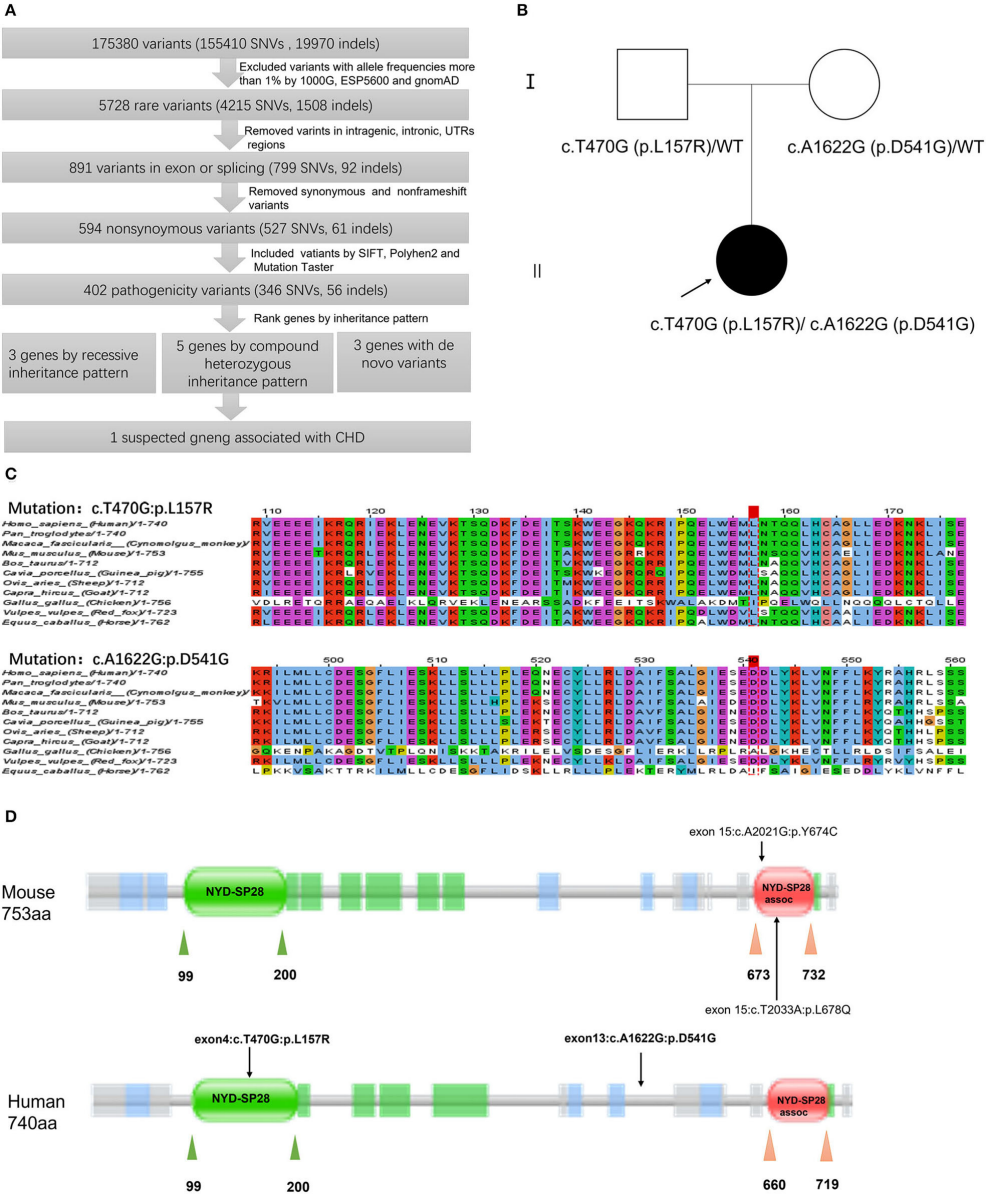
Schematic diagram of filtering strategies in our report. Several variant filtering processes and candidate gene prioritization were applied. Only one candidate gene was associated with CHD **(A)**. Pedigrees of the patient's family. The filled and unfilled symbols represent subjects with and without CHD, respectively. Arrow indicates the proband of the family. DRC1 genotypes are shown near each symbol, WT represents wide type **(B)**. Multiple sequence alignment of the DRC1 protein sequences indicates the Leu157 residue and Asp541 residue are highly conserved among various species **(C)**. Schematic of domains within DRC1 protein. Rare variants p.L678Q and p.Y674C in DRC1 had been identified in CHD mouse by Li et al. (14), while compound heterozygous variant, c.T470G (p.L157R) and c.A1622G (p.D541G), in DRC1 was identified in our patient of which c.T470G (p.L157R) variant was lined in NYD-SP28 domain **(D)**.

A total of 175,380 variants, including 155,400 SNVs and 19,970 indels, were detected and analyzed by several filtering methods (Figure 3A). Firstly, we filtered for rare variants by excluding variants with minor allele frequencies of >1% in all the three databases, including 1,000 genomic data, ESP5600, and gnomAD data (gnomAD_ALL and gnomAD_EAS), and 5,728 variants remained. A total of 891 variants were found in the exon or splice site, of which 594 were non-synonymous variations leading to amino acid alternation. Then, we applied four prediction algorithms (SIFT, Poly-Phen-2, Mutation Taster, CADD) to identify candidate deleterious variants. A total of 402 variants, predicted to be damaging by at least two algorithms, were included for further downstream analysis. Given the characteristics of the pedigree, homozygous, compound heterozygous, and *de novo* variants were considered to be a candidate for causal variations. As a next step, we prioritized candidate genes among these variants. Eleven candidate genes were determined in our patient, of which three belonged to recessive mutation, 5 compound heterozygous mutations, and 3 *de novo* mutations (Supplementary Table S2). Among the candidate genes, *DRC1* was reportedly associated with CHD (14). In our patient, the compound heterozygous variations, c.T470G (p.L157R) adopted from her father and c.A1622G (p.D541G) adopted from her mother, in the *DRC1* gene were considered as diseased causal mutations (Figure 3B).

The paternally inherited c.T470G variant, located within the exon 4 of the DRC1 gene, was found to result in substituting leucine for arginine at position 157 of the encoded protein, while the maternally inherited c.A1622G variant within the exon 13 result in substituting aspartic for glycine at position 541 of the encoded protein. The pathogenicity effect of these mutations was confirmed by prediction tools (Mutation taster, SIFT, PolyPhen-2, CADD) and both the variants were predicted to be deleterious (Table 1). Furthermore, homology analysis indicated that leucine at position 157 and aspartic at position 41 are highly conserved among various species (Figure 3C).

**Table 1:** Compound heterozygous mutation in DRC1 gene was predicted in our patient by whole-exome sequencing (WES).

**Gene name**	**DRC1**	
Genomic	2:26647252	2:26673482
Base change	T>G	A>G
Amino acid	L157R	D541G
ESP6500	.	.
1000G	.	.
GnomAD_ALL	0.00005779	0.00010593
GnomAD_EAS	0.00084836	0.00152243
SIFT	D	D
PolyPhen2	D	P
Mutation Taster	D	N
CADD	25.2	25.2
PhyloP	3.93	3.3471
PhastCons	1	1

*SIFT: D, Deleterious; T, Tolerated*.

*Poly Phen2, D-Probably damaging; P, Possibly damaging; B, Benign*.

*Mytation Taster: A, Disease_causing_automatic; D, Disease_causing; N- Polymorphism*.

*CADD: CADD Phred of >15 mean indicates the mutation is predicted to be damaging*.

*PhyloP: (values between −14 and +6) measures both slower evolution and acceleration of evolution under neutral drift; sites predicted to be conserved are assigned positive scores, while sites predicted to be fast-evolving are assigned negative scores*.

*Phastcons: Phastcons is a tool to calculate conservation score and identify conserved DNA elements with a maximum score of 1 indicating a highly conserved location on the DNA*.

*“.”Indicates the mutation cannot be predicted in the database*.

## Discussion

A hemodynamic shunt always occurs in the presence of coronary artery fistula/fistulas, whereby the blood flows from the high-pressure fistula into the low-pressure chamber. Most patients with one coronary artery fistula are asymptomatic early in life because of the small fistula with the insignificant shunt (15, 16). However, some patients who presented with large fistula/fistulas and substantial left to right shunt may have symptoms of congestive heart failure with a cardiac enlargement (7, 17–20). The latter was the case in our patient with a pulmonary to systemic blood flow ratio of 4.29, which was uniquely compounded with 4 collateral arteries to the right atrium, patent ductus arteriosus, and atrial septal defect. Congenital collateral arteries have been rarely reported (11).

Interventional treatment, catheterizations, or/ and surgery, should be considered in the case of substantial shunt, significant aneurysmal formation, or presence of other cardiac malformations (21, 22). Cardiac catheterization was firstly performed on our patient to coil-occlude two of the collateral arteries. Considering the patient's severe condition and potential contrast media overdose, together with the indications of surgery, the catheterization was stopped. Cardiac surgery was subsequently performed to repair the remaining malformations. The patient recovered well from the interventions and grew well at 3 years of follow-up.

To explore the genetic pathogenesis, WES was performed on our patient and her parents. Eleven candidate genes, including three genes with recessive variants, five genes with compound heterozygous variants, and three genes with *de novo* variants were identified in our patient. To date, about 400 genes with the causative mutation have been determined in patients or mice with CHD (13, 14, 23, 24). The genetic analysis of our patient mainly concentrated on the possibility of these known genes. Among the 11 candidate genes, the *DRC1* gene with compound heterozygous variants has been associated with CHD.

The DRC1 gene is located on chromosome 2p23.3 and contains 17 exons. It encodes dynein regulatory complex protein 1 consisting of 740 amino acids. *DRC1* is a key component of the nexin-dynein regulatory complex (N-DRC) and plays an important role in ciliary movement (25). Previous studies have reported that mutations in *DRC1* largely result in primary ciliary dyskinesia (26, 27), morphological abnormalities of the sperm flagella, and male infertility (28). Notably, a study revealed the central role of cilium and cilia transduced-cell signaling in the pathogenesis of CHD by the recessive forward genetic screen in mice. *DRC1* variants have been observed in several types of CHD in mouse models. The mouse model with recessive c.A2021G (p.Y674C) variant was presented with cardiac malformation, such as dextrocardia, atrial septal defect, ventricular septal defect, and malformation of large arteries, including interrupted aortic arch and transposition of great arteries, while mouse model with recessive c.T2033A (p.L678Q) variant was also presented with dextrocardia (14). We found both variants lined in SYD-28 assoc domain through analysis of domain by Pfam with Uniprot code Q3USS3. SYD-28 assoc is the C-terminal domain of *DRC1*. Interestingly, the c.T470G (p.L157R) variant in our patient was located at the SYD-28 domain of *DRC1*, thus, may impair the function of this domain (Figure 3D). SYD-28 is the N-terminal domain of *DRC1*. The NYD-SP28 assoc is a short region found at the very C-terminus of family members of NYD-SP28. NYD-SP28, a human sperm tail protein, is expressed in a development-dependent manner and plays a significant role in sperm capacitation (29). Presently, reports regarding the function of NYD-SP28 remain limited. Therefore, we proposed an assumption that these two domains may be partly responsible for the influence of *DRC1* on cardiovascular development. Although another damaging variant, c.A1622G(p. D541G), was located at neither the SYD-28 domain nor the SYD-28 assoc domain, it may have contributed to the functional or structural alteration of *DRC1*.

Heterogeneity is one of the major characteristics of CHD in general. A specific gene with different variants may result in different phenotypes. For example, different variants in *NOTCH1* have been observed in several types of CHD, such as pulmonary stenosis (PS) (30), left ventricular outflow tract obstructive (31), and ventricular septal defect (32). Taken together, the compound heterozygous variants, c.T470G (p. L157R), and c.A1622G (p. D541G), in *DRC1* is the most probable cause of CHD in the patient. Our study indicates the first novel variants in *DRC1* identified in humans with CHD.

The limitations of our study should be noted. WES may miss the intronic variants that are likely to have a significant role in the pathogenesis of CHD. Sanger sequencing and the animal experiment would confirm our hypothesis that the identified genetic variants contribute to the unique CHD phenotypes in our patients.

## Data Availability Statement

The datasets for this article are not publicly available due to concerns regarding participant/patient anonymity. Requests to access the datasets should be directed to the corresponding author.

## Ethics Statement

Written informed consent was obtained from the minor(s)' legal guardian/next of kin for the publication of any potentially identifiable images or data included in this article.

## Author Contributions

SS and SX has contributed to the conducting and reporting of the work described in the article. YH, JianL, and LM has contributed to the conducting of the work. XC has contributed to the planning and conducting of the research. JiaL has contributed to the planning, conducting, and reporting of the research. All authors contributed to the article and approved the submitted version.

## Conflict of Interest

The authors declare that the research was conducted in the absence of any commercial or financial relationships that could be construed as a potential conflict of interest.

## Publisher's Note

All claims expressed in this article are solely those of the authors and do not necessarily represent those of their affiliated organizations, or those of the publisher, the editors and the reviewers. Any product that may be evaluated in this article, or claim that may be made by its manufacturer, is not guaranteed or endorsed by the publisher.
